# Building adherence-competent communities: Factors promoting children's adherence to anti-retroviral HIV/AIDS treatment in rural Zimbabwe

**DOI:** 10.1016/j.healthplace.2011.07.008

**Published:** 2012-03

**Authors:** Catherine Campbell, Morten Skovdal, Zivai Mupambireyi, Claudius Madanhire, Constance Nyamukapa, Simon Gregson

**Affiliations:** a Institute of Social Psychology, London School of Economics and Political Science, London, UK; b Department of Health Promotion and Development, University of Bergen, Bergen, Norway; c Biomedical Research and Training Institute, Harare, Zimbabwe; d Department of Infectious Disease Epidemiology, Imperial College London, London, UK

**Keywords:** Antiretroviral therapy, Children, Social capital, Adherence, Social spaces, HIV/AIDS, Zimbabwe, AIDS-competent communities

## Abstract

Given relatively high levels of adherence to HIV treatment in Africa, we explore factors facilitating children's adherence, despite poverty, social disruption and limited health infrastructure. Using interviews with 25 nurses and 40 guardians in Zimbabwe, we develop our conceptualisation of an ‘adherence competent community’, showing how members of five networks (children, guardians, community members, health workers and NGOs) have taken advantage of the gradual public normalisation of HIV/AIDS and improved drug and service availability to construct new norms of solidarity with HIV and AIDS sufferers, recognition of HIV-infected children's social worth, an ethic of care/assistance and a supporting atmosphere of enablement/empowerment.

## 1 Introduction

Despite pessimistic predictions that levels of adherence to anti-retroviral therapy (ART) by HIV-infected Africans would be low, this has not been the case, with HIV positive people in many African countries achieving higher levels of treatment adherence than in North America. How have such high levels of adherence been reached in contexts of poverty, social disruption, under-resourced services and poor infrastructure? We investigate this through a case study of factors facilitating children's adherence to ART in rural Zimbabwe, with particular attention given to the social relationships – both networks and norms – with which children and their carers are located. Social relationships are central to the concept of social capital, increasingly used in debates about how to mitigate AIDS impacts in sub-Saharan Africa. Social capital has been found to impact HIV risk (Campbell et al., 2002; Gregson et al., 2004; Pronyk et al., 2008), HIV/AIDS related stigma (Chiu et al., 2008), and more recently, adherence to antiretroviral therapy (Binagwaho and Ratnayake, 2009; Ware et al., 2009; Wolff et al., 2009; Wouters et al., 2009a). Conceptualising social capital in terms of the networks and norms that characterise local communities in which children and their carers live, we examine the link between social capital and children's adherence to ART in a low income setting, and outline our evolving conceptualisation of an ‘adherence-competent community’—defined as those social relations that enable and support the likelihood of optimal adherence despite poverty and social disruption.

HIV care and treatment is complex and drug regimens must be carefully adhered to, requiring consistent and meticulous monitoring (Steele and Grauer, 2003; van Rossum et al., 2002) and the support of various actors, frameworks and systems, including the child, guardian, community members, the child's cultural heritage and the health care system available (Haberer and Mellins, 2009; Vreeman et al., 2009). It is critical that children in resource-poor settings stay on affordable, readily available and first choice treatment (first-line drugs) for as long as possible. Even though ART adherence amongst HIV-infected children in low- and middle-income countries is generally better than in high-income countries (Vreeman et al., 2008), a lack of affordable alternative medication (second-line drugs, should first-line drugs fail) means there is an even more urgent need to maximise children's adherence to first-line ART.

### 1.1 Understanding ART adherence competent community contexts

Much has been written about children's adherence to ART. However, much of this is biomedical, mostly exploring how children's ART adherence can be measured (e.g. Farley et al., 2003; Gibb et al., 2003; Nabukeera-Barungi et al., 2007; Watson and Farley, 1999), and on children in high-income countries (Simoni et al., 2007; Vreeman et al., 2008), even though 91% of all new child HIV infections occur in sub-Saharan Africa (UNAIDS and WHO, 2009). Much attention has been given to the barriers that undermine children's ART adherence, including drug palatability and formulation (Paranthaman et al., 2009; Polisset et al., 2009), poverty and stigma (Bikaako-Kajura et al., 2006) and non-disclosure of HIV status to the child (Nabukeera-Barungi et al., 2007; Polisset et al., 2009). Such papers pay less attention to how social environments can facilitate adherence. A recent pioneering study in Kenya (Vreeman et al., 2009) highlights factors including the child's age and household position, their relationship with their care giver, adult openness regarding the child's HIV status, available resources, beliefs about HIV, stigma and access to health care services. In this paper we seek to build on these findings with our Zimbabwean case study.

A literature review by Haberer and Mellins (2009) highlights that while much has been written about how child-specific factors (e.g., psychosocial function, neurodevelopment, developmental stage) and regimen characteristics (e.g., drug formulation, changes to treatment plans) may impact children's ART adherence, little is known about social factors impacting children's ART adherence. As such, our aim is to focus on social facilitators of child ART adherence. We conceptualise adherence within our wider conceptualisation of the ‘AIDS competent community’, understood as those local community resources that best facilitate effective responses to HIV/AIDS. We define the ‘AIDS competent community’ as a social setting in which people are most likely to work collaboratively to optimise HIV/AIDS prevention, care and treatment (Campbell et al., 2007; Campbell et al., 2009; Nhamo et al., 2010; Skovdal and Campbell, 2010). Even in the most resource-poor settings, communities have ‘portfolios of assets’ (Moser, 1998) which potentially serve as useful resources for public health and social development professionals seeking to optimise the use poor communities can make of prevention, care and treatment services. Social capital is one such asset and in this paper we define it as those local networks and norms which enable people to work collaboratively, in conditions of mutual trust and support, to achieve goals of mutual interest (e.g. optimal treatment of children with AIDS).

Contrary to early predictions that ART adherence in sub-Saharan African would be low due to poverty, social disadvantage, the complexity of treatment regimens and poor infrastructure (Ainsworth and Teokul, 2000; Marseille et al., 2002; Müller et al., 1998), and contrary to reviews that ART adherence is Africa is ‘often poor’ (Gill et al., 2005), it has been found that (a) high levels of adherence can indeed be achieved in poor resource settings (Coetzee et al., 2004; Orrell et al., 2003); and (b) levels of adherence are higher in many sub-Saharan African countries than in the relatively privileged North American context (Attaran, 2007; Mills et al., 2006a; Mills et al., 2006b; Vreeman et al., 2008). Using the concept of social capital, Ware et al. (2009) argue that in the United States, relative lack of supportive networks and individualistic social norms mean that people in trouble may often be isolated and unsupported. By contrast, people in Africa are more likely to look out for one another—driven by a stronger sense of collective responsibility (Ware et al., 2009). Focusing on the role of immediate and extended family in ART adherence, they argue that social capital sets up systems of ‘social coercion’. These lock ART users into circles of obligation to adhere to treatment as a sign of gratitude to kin who have made financial sacrifices to help meet their health expenses (Binagwaho and Ratnayake, 2009; Ware et al., 2009).

Our own work in Manicaland, Zimbabwe, presented in this paper, differs from Ware et al. and Bignahwaho et al. in two ways. Firstly, in line with more conventional understandings of social capital in the social sciences (rooted in Putnam, 2000; Putnam et al., 1993), we have focused not on family and kin relations, but on networks and norms in wider local community contexts. Secondly, as outlined below, we have identified very different mechanisms in explaining the impacts of social capital on ART adherence. We will highlight how, in our study, the presence of social capital served to increase peoples' sense of confidence and freedom to act in health-enhancing ways, rather than trapping them in coercive webs of social responsibility.

Against this background, we use thematic network analysis to investigate the social landscape of children's adherence in rural Zimbabwe through (i) identifying community-level relationships that assisted children and carers, and (ii) examining the social norms through which social capital impacted on adherence, against the backdrop of the coercion vs. empowerment debate we allude to in the previous paragraph.

## 2 Methodology

### 2.1 Study area and sampling

Zimbabwe transitioned from colonial to African majority rule in 1980. Soon after independence Zimbabwe experienced a GDP growth of 5%, with the introduction of free primary education and improved health services (Richardson, 2005). However, after 1999 it experienced political turmoil and a severe economic downturn, which meant that Zimbabwe's GDP declined by 8% in 2001 and 18.5% in 2003 (OECD, 2004). Although conditions have improved slightly since mid-2009, instability remains. This, coupled with the devastating impact of AIDS, has made life difficult for many Zimbabweans, with life expectancy falling from 61 years in 1992 to 42 in 2010 (WHO, 2010; ZCSO, 2007). Although the ‘natural’ epidemiology of HIV has contributed to the decrease in HIV prevalence, the decline of national HIV prevalence in Zimbabwe from 29.3% in 1997 to 16.5% in 2007 is largely explained by reductions in high-risk behaviours (Gregson et al., 2010). As a result of a peak in prevalence in 1997, many children experiencing a slow progression of HIV infection following transmission during the perinatal and breastfeeding period are now in need of ART and HIV care. In 2007 for example, it was estimated that 3.4% of children aged 10 years in Zimbabwe were HIV-infected survivors following mother-to-child transmission (Ferrand et al., 2009).

Since 2005, Zimbabwe has witnessed a gradual roll-out of ART. Using the revised 2010 WHO treatment guidelines as a benchmark – recommending initiation of antiretroviral therapy at a CD4 count of <350 cells/mm^3^ – an estimated 34% (30% for children) of those eligible for treatment in Zimbabwe were able to access the life-saving drugs in 2009 (UNAIDS, 2010). A 2008 survey of 98 HIV clinics in Zimbabwe found that 13% of all patients receiving HIV care from these clinics were between 0 and 19 years of age, of which 33% were aged 0–4; 25%, 5–9 years; 25%, 10–14 years and 17%, 15–19 years (Ferrand et al., 2010). Chief funders have been the UN-coordinated Expanded Programme of Support, financed by bilateral donors and the Zimbabwe government through the National AIDS Trust Fund, financed by a 1% tax levy ring-fenced for HIV/AIDS management.

Interviews were conducted in seven rural communities of the Manicaland province. The communities are located in or around three health facilities that provide ART services. To gather a mix of experiences, we recruited nurses and guardians receiving services from three different health facilities, namely a district hospital (with approximately 30 nurses and 2 doctors on duty during the day), a large mission hospital (with approximately 30 nurses and 1 doctor on duty during the day) and a rural mission health clinic (with 2 nurses on duty during the day and with a doctor visiting a couple of times a week).

All the communities are crippled with poverty and livelihoods are primarily sustained through subsistence farming. Only few have excess produce to sell at market centres. Although formal employment is limited, some men work in a large forestry estate. Others migrate to cities to seek work. People struggle to spare even a dollar to pay the regular consultation fee to receive ARV treatment. This is recognised by various international organisations who provide food aid to improve patients' spending power for health care and supplement their diets.

Many obstacles stand in the way of effective HIV care and treatment in Zimbabwe. Our own work in Manicaland, and indeed that of other researchers, has highlighted that some patients struggle to adhere to treatment because of associated costs (Muchedzi et al., 2010; Skovdal et al., 2011c), limited support from family members (Skovdal et al., 2011b, d), and conflicts and frustrations arising from differences between local realities and biomedical expectations (Campbell et al., 2011a, b; Skovdal et al., 2011a). Poor infrastructure, shortage, or inadequate training of health staff (Cooper, 2010; Skovdal et al., 2011c), lack of malnutrition services for HIV positive people (Prendergast et al., 2011) as well as poor referral services (Muchedzi et al., 2010) are some of barriers to quality care and treatment in Zimbabwe.

Despite the dramatic political and economic uncertainty of the past decade, disruption to HIV services due to political crackdowns by central government, and highly uneven provision of public services (Amon and Kasambala, 2009), Zimbabwe has scored unexpectedly highly on some indicators of health and well-being relative to other southern African countries: contraceptive use (ZCSO, 2007), ART coverage (UNAIDS, 2010) and HIV-avoidance (Gregson et al., 2010), even with significantly less external funding for health (APHA, 2010). ART adherence is better in Zimbabwe than in some of the more stable countries in sub-Saharan Africa, such as Tanzania and Mozambique. As such, it provides a fascinating arena for a study of local people's capacity to mobilise their own indigenous resources to respond to social problems, often without the outside interventions and funding characterising health and social development efforts in many other African settings.

We draw on perspectives of 25 nurses and 40 guardians of children on an ART programme. Guardians were sampled using snowball (via village community health workers), opportunistic (self-selected informants) and typical case (adherers to ART) sampling. Nurses from the three rural health facilities were recruited on the basis of their willingness to participate. Our study is hosted by the Biomedical Research and Training Institute (BRTI), the National Institute of Health Research (NIHR) and the Ministry of Health and Child Welfare (MoHCW) of Zimbabwe.

Ethical approval was granted by the Medical Research Council of Zimbabwe (A/681) and Imperial College London (ICREC_9_3_13). Participants gave written informed consent to participate, under conditions of anonymity and confidentiality, with the option of withdrawing their participation at any time. Pseudonyms are used below.

### 2.2 Data collection and analysis

Data were collected between October 2009 and March 2010 by four experienced fieldworkers, all qualified social workers, comprising 39 in-depth interviews and 3 focus groups in the Shona language. Semi-structured topic guides, covered informants' personal backgrounds, experiences of AIDS, stigma and ART treatment, and factors facilitating or hindering children's ART adherence. Interviews lasted an average of one hour; focus groups two hours and twenty minutes.

Interviews were digitally recorded, transcribed verbatim, translated into English and imported into Atlas.Ti for analysis (Flick, 2002). We used Attride-Stirling's (2001) four-step thematic network analysis: coding text segments with an interpretative title (step 1); exploring links between codes by identifying dominant themes (relating to social determinants of children's ART adherence and psych-social mediators) (step 2); these were clustered into higher order ‘organising themes’ (step 3). These were further clustered into the over-arching global theme reflecting our research question in this paper: what social factors facilitate children's adherence to ART in Zimbabwe? We use the structure detailed in Table 1 to present our answers to this question.

Given that participation was voluntary, with recruitment from clinic settings, our sample was biased towards regular, highly motivated child carers, who had overcome multiple barriers to access and adherence (discussed below). Furthermore, rather than seeking to generate stereotypical characterisations of all children with HIV and their carers, we have sought to map out some of the diverse ways child adherence has been facilitated in one setting.

## 3 Findings

After providing a brief account of barriers to adherence, we use the distinction between network and norm dimensions of social capital as the frame for our account of the social relationships that facilitated child adherence.

### 3.1 Barriers to adherence in Manicaland

Although this paper highlights factors that sustain children's adherence, as already stated, this should not overshadow the multiple obstacles faced by Zimbabwean ART users of all ages: lack of food, distance to health clinics, transport and opportunity costs, and high clinic attendance fees—US$1 per month where the average person lives on less than $2 a day (Skovdal et al., 2011c). Adherence is often inhibited by stigma (Campbell et al., 2011c), social constructions of masculinity, which interferes with both men and women's ART adherence (Skovdal et al., 2011a, d), as well as fears of disclosing HIV status to friends and family and lack of support from family and community (Skovdal et al., 2011c). At an institutional level, poor services, long waiting times, impatient or unsympathetic nurses and poor communication between service users and providers also deter some ART users from adhering to treatment plans (Campbell et al., 2011a, b).

Some obstacles are unique to children's ART adherence. These include the barriers related to the age and physical and mental capabilities of some guardians (Skovdal et al., 2011b). Elderly guardians were more likely to live in poverty. Immobility, deteriorating memory and poor comprehension of complex treatment regimens meant some battled to ensure optimal adherence by children in their care (Skovdal et al., 2011b). Immobility and distance to health facilities prevented some from attending monthly consultations, crucial for the optimal ARV monitoring and distribution. Some guardians forgot to dispense drugs when the child appeared healthy—frustrating nurses and compromising the quality of important guardian-nurse relationships (Campbell et al., 2011b).

Nevertheless, nurses generally commented that despite these difficulties, child adherence tended to be good. We now examine social factors facilitating such adherence.

### 3.2 Factors facilitating children's adherence to ART in Manicaland: networks

#### 3.2.1 Community understanding and support

The first factor perceived to impact on adherence was a gradual increase in community support for AIDS-affected families. Whilst stigma had originally served as a severe barrier to service access and treatment adherence, and still did for some people (Nhamo et al., 2010), others spoke of the gradual normalisation of AIDS, as AIDS-related knowledge and first-hand experience of family or friends with AIDS increased. Informants said this had gone hand in hand with increased community acceptance of the inevitable role every single person had to play in responding to this epidemic—including contributing to the care and support of AIDS-affected children.

“In my area lots of people are now informed about HIV and most are willing to look after AIDS-affected children, only a minority would refuse—people now realise HIV has become a national problem and almost everyone is affected.” Loyce, age 42, cares for her 9-year-old nephew who is HIV positive and orphaned. Loyce lives with her own child, one niece and three nephews.

The second factor serving to mitigate stigma was the availability of life-prolonging medication, severing the AIDS-death link in the public imagination. This provided caregivers with the possibility of offering effective care, and showed the world that HIV positive people could live relatively normal lives and deserved to be treated normally.

“There is a huge difference now—if an HIV positive person eats off a plate and I wash it and give it to the next person, they will be willing to eat off it.” Sandra, age 59, cares for her 16-year old granddaughter who is HIV positive and orphaned.

Such changes in attitude made it easier for carers to fulfil their commitments. Religious representations also informed many informants' accounts of how they responded to HIV/AIDS. Guardians of HIV-infected children frequently commented that God would reward their commitment.

“It is good to look after HIV-infected children, it blesses, and it brings you blessings from God.” Hilda, age 39, cares for her 10-year-old niece who is HIV positive and orphaned.

The fact that caring for HIV-infected children is seen as commendable makes it easier for guardians to negotiate material and financial support from other community members—neighbours, relatives, fellow church members. Many guardians spoke of borrowing money to cover medical expenses.

“The consultation fee is affordable. Even if you do not have money, you can easily ask a fellow community member for a dollar, and repay them later.” Carolyn, age 40, cares for her 7-year old niece who is HIV positive and orphaned.

However, a few guardians were reluctant to borrow, saying the cost of monthly consultations continued to represent a major barrier.

“Finance is a major factor. It is not good to always borrow money that you struggle to pay back—so you have to look for the money first.” Cephas, age 40, cares for his 15-year-old nephew who is HIV positive and orphaned. He lives with his wife and two other children.

#### 3.2.2 NGO activities

Informants spoke of three ways NGOs had facilitated adherence. The first was their contribution to educate people about HIV and AIDS, contributing to stigma reduction.

“Some think if children on ART play with their children they might infect them. Look at Gilbert—those white marks all over his body. Some might not want him near their children. But these are only a minority. Most are now enlightened about how HIV is spread, well informed through awareness and educational campaigns. A lot of organisations have been teaching about HIV/AIDS.” Loyce (see above).

Thus, whilst there still is a stigmatizing ‘ignorant’ minority, NGO campaign efforts appear to be trickling through. The second pathway through which NGOs have facilitated an adherence-competent context was through their child-centred and community-based counselling and HIV testing programmes.

“We got to know about the child's status through the Mirdza programme running in our area, we were told about the counselling and testing for children at the school in September.” Marjorie, age 37, cares for her 7-year-old nephew who is HIV positive and orphaned. She lives with her three children and the nephew.

The third pathway was through NGO-distributed food parcels to households with members on ART. These were particularly helpful for HIV-infected children living with elderly guardians in poverty.

“Some children are cared for by old grandparents so they lack food—but now that problem has been met because a lot of organisations, such as CAREAF, are distributing food in the area.” Marie, age 36, nurse working in a voluntary counselling and testing centre.

NGO help was not always uncomplicated however. While some guardians received NGO food through referrals by the child's doctor, others said that to qualify they had to attend a community meeting where the HIV-infected needing food were asked to raise their hands—with fear of stigma often making carers reluctant to disclose the child's status in public.

#### 3.2.3 Accessible health services

Due to the complex nature of ART and potential side-effects, children often required careful monitoring by health professionals, making the availability and quality of health services essential for optimal drug use. The ART roll-out in Zimbabwe is well under way and has led to the strengthening of HIV management services. Although much work remains to be done in de-centralising ART services, great efforts have been made to install and run CD4 count machines (to determine the stage of HIV progression and treatment efficacy) at district-level health facilities – something which other countries (e.g. Malawi) in southern Africa have been struggling with (Makombe et al., 2006). Several informants spoke of how strengthened services had facilitated child adherence.

“This ART programme has been very effective as many now know they can access ARVs for free. People have just been worried about the CD4 cell tests, which we used to send to (the nearest city) before we got the machine. So patients would have to come twice or more before they could have their results processed and sometimes blood samples had to be taken twice or more as well. Some threatened to drop out. But since we had the CD4 machine here we have been running smoothly. We used to do CD4 tests once a month but now we do once a week.” Carl, age 56, has been working as a nurse for 30 years.

Having said this, the CD4 count machine in one of the hospitals in our study area was said to be infrequently serviced, with recurrent break downs. Furthermore, a minority of informants reported periodic shortages of ARV drugs. Most had not yet experienced shortages however.

“At our hospital we have been blessed. We get ARTs for free and we have never run short of them—they are available each time we go to collect our monthly supplies.” Loyce (see above).

Regular ARV availability is not only important for user health. It also builds both user and provider trust in health services, opening possibilities to transform HIV into a chronic illness rather than a death sentence. It has also renewed the motivation of previously demoralised nurses, now able to play an effective role in prolonging HIV-infected children's lives.

“I have a great sense of achievement when I see how children and guardians comply with ARVs and the child's health improves. I also feel as if I am able to do something good for other people.” Ropafadzo, age 56, is a nun who has worked as a nurse for 20 years.

Several nurses spoke of how the renewed impetus and sense of effectiveness inspired them to take their jobs more seriously, improving patient/guardian-nurse relationships, leading to better adherence. A number of guardians spoke of the competence of the nurses, as well as their respect and caring for their patients.

“What I am grateful about, since my child hasn't been feeling well, is the way they talk to me there at the hospital. I am happy with it. They make thorough investigations of the history of the child's illness, they ask how the child has been faring, what the problem is, what are the presenting symptoms and everything, I am very impressed by the way they treat me […] I was happy with the whole process of blood testing for our children, the nurse talked to me with warmth and love, she instructed me politely on how to administer the tablets, she advised me and asked me how I was going to disclose the status to my child when she is of age. I told her that my relationship with the child will facilitate disclosure when the time comes, that's what impressed me.” Carolyn (see above).

Health service staff were said to actively engage with guardians and treatment partners to ensure they were fully equipped, emotionally and practically, to facilitate adherence. This included establishing support groups for guardians and counselling on how to look after a child on ART.

“When you get to the hospital and you are done with treatment issues then you go for lessons on how to look well after the child, I think I can say it helps us a lot in my life” Hilda (see above).

There is little doubt that improvements in HIV management (albeit patchy in some cases) have strengthened guardian's trust in the health services in ways that have facilitated the likelihood of optimal child access and adherence to ART.

#### 3.2.4 The role of treatment partners

A few nurses spoke of child carers taking advantage of the orphan status of HIV-infected children, forcing them to do heavy duties that compromised their health and response to drug treatments. However, all the carers in our study spoke of their love and tolerance for the child in their care and the importance of not treating children on ART differently from other children.

“Yes we do face challenges as we care for the children but we should facilitate their treatment and care for them the same way we do to our own children. Even my own family can be a burden so we should not discriminate these children because they are HIV positive.” Cephas (see above).

Nurses repeatedly highlighted the importance that children on ART had a permanent ‘treatment partner’, preferably someone who lived in the same house. Child carers had a key role to play in working with nursing staff to facilitate and monitor medication.

“We need to establish who exactly is staying with this child, and who will continue to take care of them. Even with adult patients we need to establish a treatment buddy. We cannot take compliance for granted; we cannot give drugs to a person who is staying by herself. What if she fails to take the tablets, who will tell us? If the patient forgets to turn up for monitoring, who will remind them? We need to have a point of contact, someone who will look out for the patient, which is the treatment buddy.” Nicole, age 34, is a head nurse. She has been working as a nurse for 13 years.

Child carers were keen to demonstrate their knowledge about paediatric ART and show their dedication and commitment to the child, emphasising that a ‘good’ carer understands the importance of nutrition and timely medication, and the importance of seeking urgent medical help should complications arise.

“Children on ART need enough food and a balanced diet […] We make sure they take their drugs all the time. We were taught that this treatment is for life—so we were told that you choose your favourable hour and you keep to that because if you do not keep regular times resistance might develop.” Cephas (see above).

“I rush to the clinic at the slightest sign of sickness. I first go to my nearest clinic that is at Samachina. If they refer me to Dabon and if there is still time I rush, but when it is late I go the next day.” Marjorie (see above).

The unpleasant taste as well as the formulation of ARVs makes it challenging for some child carers to persuade children to take them. They needed to adopt various strategies to overcome such hurdles, for example ‘bribing’ children with gifts or treats, such as juice (an exceptional treat in an impoverished environment).

“You tell the child: “if you take the medication I will give you some juice”. They will agree, they will definitely be motivated to take their medication. You do not realise how much trouble we are going through with these children.” Violet, age 43, cares for an 8-year-old child from the community who is HIV positive and orphaned.

#### 3.2.5 Children's active participation

NGO and hospital counsellors encourage child carers to overcome their tendency towards denial and their fear of stigma, and tell the HIV-infected child why they are on medication. A growing number of child carers (though not all) do indeed tell the children about their HIV status, providing children with the opportunity to work as genuine treatment partners with their carers to achieve optimal adherence. However, regardless of whether full disclosure of the child's HIV status has taken place, children and their carers are both required to attend monthly review consultations to assess the child's progress and collect their monthly supply of drugs. Although parents find it difficult to tell their children that they are HIV positive (Brown et al., 2011), there are benefits for children to know their HIV status, including their psychological adjustment (Bachanas et al., 2001) and adherence to ART (Bikaako-Kajura et al., 2006; Haberer et al., 2011). We found that a child who is aware of their status is more likely to appreciate the importance of ART adherence, and more likely to take an active role in following advice given during monthly consultations.

“My child knows the importance of these drugs and remembers his review dates well.” Nyasha, age 42, cares for her 9-year-old son who is HIV positive. She lives with her husband and three other children.

“Sometimes the child would be the one reminding the care giver. The good thing about well-informed children is that they are more likely to remember all we tell them and they do exactly what we have advised. They will never forget their review dates and when they come on that date, the care giver will say they have been reminded by the child. Sometimes it's the child that is the one who remembers every time. And some of these children participate very well in support groups.” Carl (see above).

For some children, taking medication becomes a habit, part of their everyday lives.

“My child is still very young, initially in the early days, she would refuse, I would have to persuade her. Nowadays she is jealous about being seen to be the one who remembers. When its dusk she will remind everyone, ‘I did not take my medication mum, I did not take my medication’—she remembers even when I am not there.” Janet, age 38, lives with her 4-year-old daughter who is HIV positive.

Children's active participation in ART adherence compliments their treatment partners' efforts very well, particularly if their carers are elderly and struggle to remember treatment details.

### 3.3 Factors facilitating children's adherence to ART in Manicaland: norms

As discussed above, much existing research has focused on *barriers* to children's adherence. In this paper we have focused on *facilitators* of adherence, particularly in the light of higher than expected levels of ART compliance witnessed in African settings. In many ways, the context of ART use in Manicaland remains unstable, with uncertainty about the supply of drugs, the future of the health services, poverty and political strife contextualising peoples' efforts to ensure the well-being, even survival, of children living with HIV. The aim of our paper has been to look at how remarkable levels of child adherence may be achieved, even in such challenging circumstances.

Above we have outlined our informants' accounts of how the inter-linked phenomena of (i) gradual public acceptance of HIV/AIDS (in the face of initially strenuous denial) and (ii) increased health service effectiveness associated with treatment availability have created a favourable climate for ART adherence. We have outlined five networks (community, NGOs, service providers, the guardians and children themselves) that have facilitated adherence within the context of these two favourable developments, even in wider social contexts of great economic and political uncertainty. To identify the interactions between these networks, we sought also to explore the social norms inherent within them. Column 3 of Table 1 above listed the social norms inherent in the relationships facilitating adherence. Table 2 provides a further analysis of these norms to provide a more detailed analysis of the ART-related empowerment they facilitated.

Our analysis suggests that – in the contexts of reduced stigma and increased treatment availability – the emotional, practical and material support inherent in the actions of the five adherence-enhancing groups outlined above perpetuated norms of solidarity with affected children, a recognition of their social worth, an ethic of care and assistance towards them, and an enhancement of the agency of both children and those concerned with their well-being. Contrary to Ware et al. (2009) and Binagwaho and Ratnayake's (2009) account of ‘social coercion’ as the mechanism mediating between social capital and ART adherence, our research suggests that social capital impacted on adherence through norms associated with enablement and empowerment rather than negative socio-emotional pressure.

## 4 Conclusion

We have highlighted aspects of the interface between service users and service providers in the context of ARV therapy in rural Manicaland, with particular attention to the networks and norms that facilitate an optimal ‘fit’ between patient and treatment, particularly remarkable for their achievement in social settings characterised by great political and economic uncertainty. HIV and AIDS services are most likely to succeed if they identify and facilitate the local community resources most likely to enable such a fit. We have highlighted the nature of the local social relationships that figured prominently in guardians' and nurses' account of factors shaping child adherence. Furthermore, contrary to similar studies in Sub-Saharan Africa, we argue that, in our context at least, social capital has worked through creating a sense of empowerment, enablement and confidence amongst children, guardians and service providers.

We argue that programmes seeking to facilitate optimal adherence are most likely to succeed if they facilitate the positive norms outlined in Table 2 above. Experiences elsewhere suggest that NGOs can play an important role in facilitating empowering norms of this nature. In Khayelitsha, South Africa, for example, the awareness campaigns and service provisions of multiple NGOs in the area have de-mystified HIV, encouraging testing and contributing to lowered HIV prevalence (Levy et al., 2005). Although time, people's experiences and multiple HIV programmes can gradually facilitate a de-mystification and normalisation of AIDS in the public sphere, a more concrete intervention that can initiate or capitalise on such a social change and strengthen social capital is that of social action funds (Glenn, 2009; Skovdal, 2010; Skovdal et al., 2010), in the form of monetary allocations to community groups who have formulated a plan of collective action to tackle obstacles faced by vulnerable households within their community. Our findings also accentuate the importance of a comprehensive health care system, staffed by motivated and confident personnel. Reflecting observations made by Stein et al. (2007) in South Africa, the findings presented in this paper suggest that ART availability can empower and restore the agency and motivation of nurses, previously demoralised by the emotional drain of dealing with terminally ill patients for whom little could be done. Such a change can influence interaction between service users and providers, which in turn influence the receptiveness (and thereby adherence) to services by users. Likewise, a trust and confidence in the availability and quality of ART services by users is equally important in motivating adherence to ART. Particularly noteworthy in our study is evidence for the integration of NGO activities with health services. For example, ART users with a patient card issued by the local health clinic would qualify for food aid from local NGOs. Similarly, upon the recommendation of the local health services, NGOs took an active role in establishing social support groups for child carers and adult ART users—an activity, which has also proved very efficient in achieving successful ART adherence amongst adults in South Africa (Wouters et al., 2009b).

To conclude, this paper has identified five sets of key social actors in promoting adherence competent contexts for children on ART: children themselves, their guardians, community members and the external agencies – including both health service providers and NGOs – that provide invaluable services to the children and their guardians. Programme planners and policy makers must pay particular and systematic attention to how best to empower these social groups in the interests of developing ‘adherence competence’. Based on our findings in the specific context of children's adherence in rural Zimbabwe, we propose a conceptualisation of an ‘adherence competent community’ as a social landscape where local community members, nurses, NGOs, guardians and children themselves are able to optimise opportunities that have arisen from (i) the gradual normalisation of AIDS in public sphere; and (ii) improved drug and service availability to work collectively to promote optimal child health through

• promoting solidarity with children and carers,
• recognising the social value of children with AIDS, their carers, and the activity of caring,
• promoting an ethic of assistance and
• restoring a sense of agency and confidence through recognising and enhancing the competence of nurses, carers and children themselves.

Clearly this is a small-scale qualitative study, conducted in one particular country; at a particular moment of the HIV epidemic and ARV roll-out. We encourage colleagues to use this framework to engage in further exploration of the community-level facilitators of ART adherence in other contexts, and in larger scale studies, to challenge or corroborate our conceptualisation.

## Figures and Tables

**Table 1:** Coding framework: social facilitators of adherence (networks and norms).

**Codes**	**Basic themes: social relationships facilitating adherence**	**Basic themes: social norms inherent in these social relationships**	**Organising themes key networks**	**Global theme**
– AIDS part of public sphere – Knowledge, attitude and behaviour change – Less stigma – Friendship building – Pious links with community recognition – Can borrow money	(1a) Improved knowledge of HIV/AIDS and more widespread understandings of PLWHA encourage people to care for infected children.	(1b) Solidarity with infected children; Recognition of children's social value; Recognition of children's right to care; Ethic of assistance for HIV-infected	**Community** understanding and support	Social factors sustaining children's adherence to ART
	(2a) Declining stigma makes it easier to provide care and support for a child with HIV, enabling children to live normal lives and develop supportive friendships.	(2b) Normalisation of children with HIV and AIDS in the community; Solidarity with infected children		
	(3a) Communities recognise role of guardians	(3b) Solidarity with guardians; Recognition of value of HIV care and support.		
	(4a) Some guardians able to borrow money to overcome economic obstacles	(4b) Solidarity with carers and children; Ethic of assistance for HIV-infected		
– External change agents – Food aid	(5a) NGOs play important role in disseminating knowledge of HIV/AIDS	(5b) Solidarity with HIV-infected people; normalisation of HIV/AIDS (vs. othering)	**NGO** activities facilitate social change	
	(6a) NGOs help mobilise social support groups	(6b) Solidarity amongst child carers		
	(7a) ARV users access food packs from NGOs	(7b) Ethic of assistance for HIV-infected		
– Improved ARV access – Paediatric services – Nurse motivation – Satisfaction with health services – Benefits of counselling	(8a) ARV services have improved with more CD4 count machines available and readily available drugs.	(8b) Restoration of agency to hospital	Improved, accessible **health services**	
	(9a) Nurses motivated by availability of life-prolonging drugs, improving nurse-patient/guardian relationships.	(9b) Restoration of nurses’ agency; Solidarity amongst nurses and patients		
	(10a) Guardians satisfied with service from the health facilities.	(10b) Enhancement of guardian's agency/confidence in their ability to provide adequate care		
	(11a) Guardians provided with counselling to become good treatment partners for children and to ‘accept’ the child’s HIV status.	(11b) Enhancement of guardian's agency/confidence to provide adequate care; Enhancement of guardian competence		
– Treatment partner – Supportive guardians – Guardians have good HIV/ARV knowledge – Guardians follow instructions – Guardians support each other – Incentivize children	(12a) Guardians accept and treat the child as their own	(12b) Enhancement of guardian commitment; Solidarity between guardians and children	**Guardian's** role as treatment partners	
	(13a) Guardians able to be reliable treatment partners.	(13b) Enhancement of guardian confidence; Enhancement of guardian competence		
	(14a) Guardians have adequate knowledge about AIDS and ARVs; follow nurses’ advice.	(14b) Enhancement of guardian confidence; Enhancement of guardian competence; Solidarity between guardians and nurses		
	(15a) Guardians group together and support each other in addressing challenges.	(15b) Solidarity amongst guardians		
– Health improvements – Agency of children – Follow instructions – Attend review dates	(16a) Children understand their condition; understand importance of drugs.	(16b) Enhancement of children's competence; enhancement of children's agency	**Children's** agency and participation	
	(17a) Children see drugs as habit, taking them a game	(17b) Enhancement of children's agency		
	(18a) Children follow instructions, remind guardians to dispense them, and attend review dates.	(18b) Solidarity between guardians and children; enhancement of guardians and children's agency		

**Table 2:** Social norms mediating between networks and adherence.

**Basic themes**	**Organising themes**
• Community solidarity with guardians • Community solidarity with children • Solidarity between guardians and children • Increased guardian commitment to children • Solidarity between nurses and patients • Solidarity amongst guardians themselves	Solidarity

• Recognition of children with HIV (as normal kids—as part of the normalisation of HIV, they were less likely to be seen as ‘other’) • Recognition of children's right to take control over own health • Recognition of the value of caring as an activity	Recognition of children's social value

• Commitment of children's treatment partners • Support by community members • Assistance available from NGOs • Co-operation between NGOs and health facilities for improved care	Ethic of care and assistance

• Restoration of nurses role to save lives • Restoration of hospitals as health care providers • Enhancement of guardian's agency—both in relation to their competence and confidence • Enhancement of children's agency	Enhancement of agency and empowerment
